# Spontaneous Resolution of a Confounding Insular Lesion

**DOI:** 10.7759/cureus.2053

**Published:** 2018-01-11

**Authors:** Ali S Haider, Christopher S Graffeo, Avital Perry, Lucas P Carlstrom, Terry C Burns

**Affiliations:** 1 Texas A&M College of Medicine; 2 Neurologic Surgery, Mayo Clinic

**Keywords:** differential diagnosis, insula, infection, resolution, glioma, abscess, imaging

## Abstract

Insular gliomas were previously considered inoperable lesions and were typically treated via biopsy, chemotherapy and/or radiation, if not observation alone. Stereotactic biopsies of low grade insular gliomas can underestimate tumor grade or fail to establish malignancy. Moreover, the survival advantages of maximal safe resection for insular lesions are increasingly being recognized. As such, early surgical resection is increasingly being performed. As with most lesions, a differential diagnosis exists for apparent insular gliomas, with definitive diagnosis generally obtained upon resection. We report an illuminating case that presented similarly to an insular glioma undergoing malignant transformation, but resolved spontaneously following a nondiagnostic biopsy. A 53-year-old female patient presented with aphasia and dizziness, followed by syncope and a 30-minute loss-of-consciousness. Imaging findings included a 12 mm region of contrast enhancement and central necrosis within a larger 3.5 cm expansile, T2-hyperintense lesion involving most of the left insula and extending into the anterior left temporal lobe. Imaging was felt most consistent with high-grade glioma. Stereotactic biopsy demonstrated nonspecific gliosis without definitive neoplastic tissue. A systemic workup was unrevealing, and thus an open procedure was subsequently planned in the intraoperative magnetic resonance imaging (MRI) suite. Preoperative MRI demonstrated interval resolution of the original lesion, despite profound T2 hyperintensity along the needle tract; thus, the planned resection was aborted. Subsequent imaging and systemic studies failed to establish a definitive infectious, neoplastic, autoimmune, or other diagnosis. However, poor dentition, history of a recent dental procedure, and the tiny central focus of diffusion restriction on the index MRI rendered abscess the most parsimonious explanation. On follow-up imaging, the lesion was noted to have further resolved without intervention. Our case illustrates the complexity of managing insular lesions and highlights the potential for alternate pathologies that can mimic insular glioma. Additionally, it provides a humbling reminder that, even in the presence of seemingly pathognomonic imaging findings, a differential diagnosis of insular lesions must be thoughtfully considered in patient counseling and presurgical planning.

## Introduction

Insular gliomas represent a uniquely challenging subset of primary brain tumors, which typically arise from white matter tracts adjacent to the mesocortex or allocortex [1]. This perimesocortical structure is notorious for its complex anatomy and elevated surgical risk due to the adjacent lenticulostriate perforators, middle cerebral artery (MCA) branches, and eloquent white matter tracts. Magnetic resonance imaging (MRI) is the diagnostic workhorse for lesions of the insula, due to its robust characterization of anatomic detail and key tumor features, including T2 hyperintensity, variably elevated perfusion, and less frequent contrast enhancement or necrosis [2]. When present, contrast enhancement raises concern as a harbinger of malignant transformation, as may develop if such lesions are merely observed [3].

In most supratentorial locations, MRI adequately distinguishes tumor from abscess; however, in the compact and anatomically complex insula, edema surrounding an abscess or metastatic lesion may masquerade as the typical T2 hyperintensity of a glial tumor, creating a diagnostic dilemma [4]. Given the frequency of false negative specimens taken from this region, biopsy is increasingly favored only by those who consider the insula “inoperable,” with others preferring to maximize tissue sampling at time of open surgical resection. The survival benefit of maximal resection for insult gliomas, when safely performed, has been increasingly recognized [1]. As such, surgery for an insular glioma may increasingly be considered in the setting of an equivocal diagnosis. We report here an unusual and interesting case of a patient who presented with insular T2 hyperintensity and spotty enhancement most consistent with a left insular glioma undergoing malignant transformation, which was subsequently suspected to be abscess and ultimately resolved after a nondiagnostic biopsy.

## Case presentation

A 53-year-old woman with no prior neurological history presented to the emergency department after an isolated episode of word finding difficulty and dizziness, followed by syncope and a reported 30-minute loss-of-consciousness. No tongue biting, bowel/bladder incontinence, or other sign of ictal activity was observed, and she recovered to her neurologic baseline. Her medical history was significant for occasional nighttime headaches—severe enough to wake her from sleep—and chronic right-sided hearing impairment associated with prior chronic otitis media status post mastoidectomy. Her physical exam was unremarkable, with no other focal neurologic deficits; her family history was significant for malignancy in two second- and third-degree relatives.

**Figure 1:**
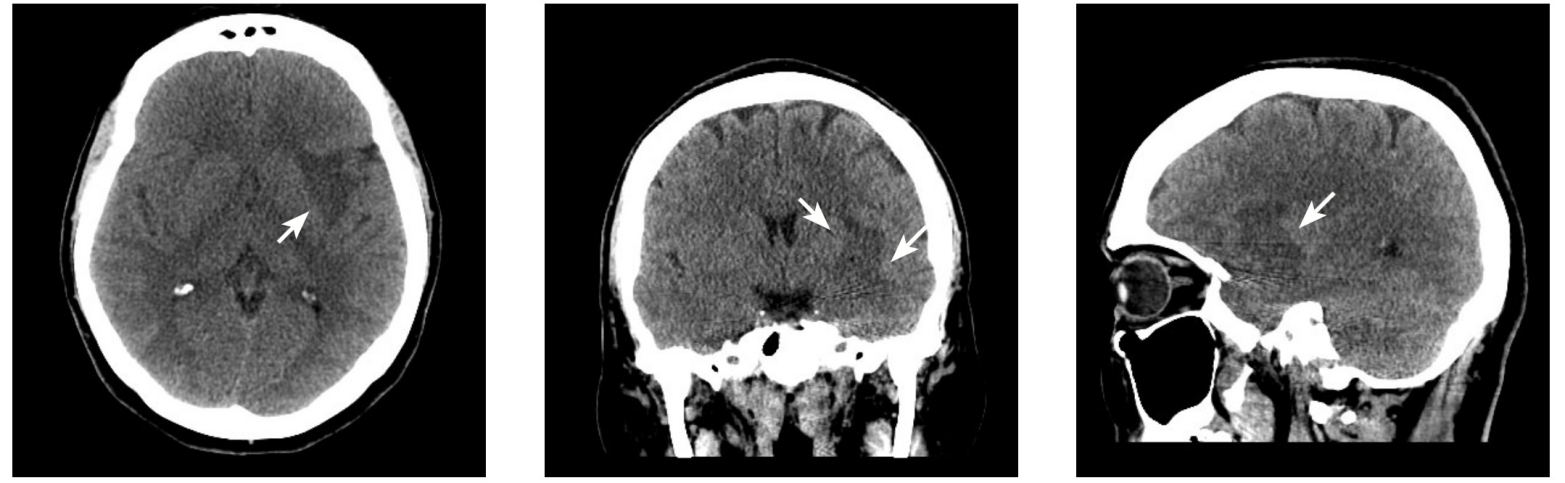
Axial computed tomography indicative of vasogenic edema in the left anterior perisylvian region (arrows).

A head computed tomography (CT) demonstrated low attenuation changes suggestive of vasogenic edema in the left anterior perisylvian region, with loss of insular grey-white differentiation and ambiguous mass effect (Figure 1).

**Figure 2:**
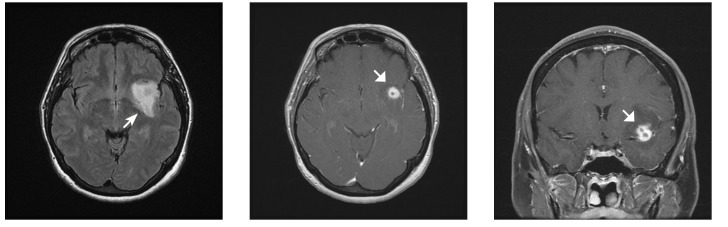
Axial magnetic resonance imaging suggestive of insular glioma, with concerning features including confluent, anatomically restricted fluid-attenuated inversion recovery hyperintensity (arrow) and nodular central enhancement with probable areas of internal necrosis (broad arrows).

A follow-up MRI demonstrated a 3.5 cm expansile, T2-hyperintense lesion involving most of the left insula and extending into the anterior left temporal lobe, within which was noted a 12 mm region of contrast enhancement and central necrosis. The imaging findings were felt most consistent with high-grade glioma (Figure 2).

**Figure 3:**
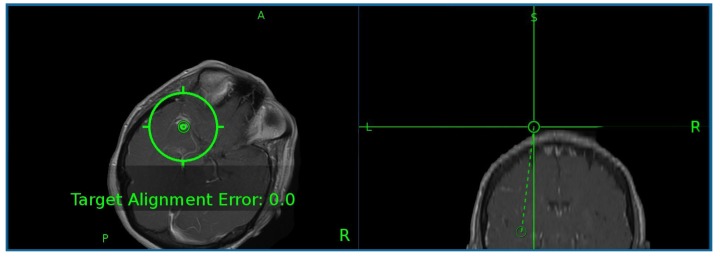
Intraoperative magnetic resonance imaging pinpointing focus of maximal enhancement for stereotactic biopsy planning.

Levetiracetam 750 mg BID PO and dexamethasone 1 mg q4h PO were initiated for seizure prophylaxis and perilesional edema, and a stereotactic biopsy was subsequently performed. Intraoperatively, the focus of maximal enhancement was targeted, and the operation proceeded uneventfully (Figure 3).

**Figure 4:**
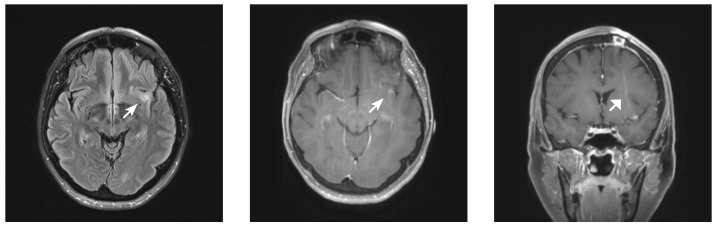
Magnetic resonance imaging demonstrating complete resolution of previously noted contrast enhancement, within a markedly reduced region of insular T2 hyperintensity (arrowheads); of note, a strip of expected T2 hyperintensity was noted surrounding the needle tract, excluding a technical error underlying the signal change at the lesion (broad arrow).

The final pathology identified reactive gliosis and diffuse, nonspecific histopathologic abnormalities, without definitive neoplastic features. The systemic workup was unremarkable, including blood cultures, serum C-reactive protein (CRP), erythrocyte sedimentation rate (ESR), echocardiogram, and cerebrospinal (CSF) analysis. The microbiology testing of the biopsy specimen was also negative, including Gram, mycobacteria, Nocardia, fungal, and acid-fast bacillus stains. Given the nondiagnostic biopsy and confounding clinical course, the case was referred to our surgical neuro-oncology team and presented at a multidisciplinary conference, from which we recommended awake craniotomy in the intraoperative MRI suite to obtain tissue diagnosis and potentially proceed with maximal safe resection, as indicated. The patient consented to awake craniotomy in the intraoperative MRI suite. However, the preoperative MRI failed to demonstrate the substantial previously observed insular T2 hyperintensity despite new significant fluid-attenuated inversion recovery (FLAIR) signal surrounding the needle tract. The prior area of contrast enhancement was similarly greatly reduced (Figure 4).

**Figure 5:**
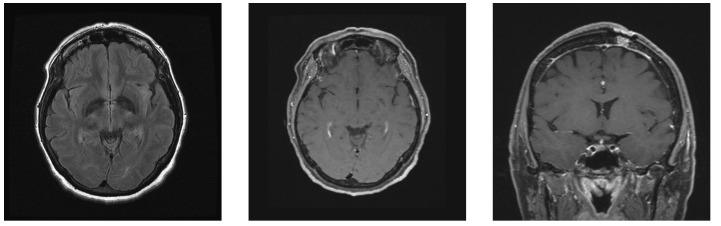
Magnetic resonance imaging demonstrating further lesion resolution with a return to near-normal regional anatomy.

Given these imaging findings, the possibility of insular glioma was now felt to be decidedly unlikely. Although the still-awake patient was prepped in pins for the procedure, a mutual decision was reached to abort the case. All further studies failed to establish a definitive diagnosis; however, in light of what was subsequently determined to be extremely poor dentition and a history of recent self-extraction of a tooth, as well as a small central focus of diffusion-restriction on the original MRI, an abscess was thought to be the most likely diagnosis. The patient elected to observe the lesion, and follow-up MRI at three months demonstrated further resolution of the lesion with overall restoration of normal regional anatomy (Figure 5).

As of last follow-up at six months, the patient has remained completely asymptomatic with no evidence of ictal activity, paroxysmal spells, or other neurologic abnormalities, and she has displayed continued radiographic stability and no clinical evidence indicating an underlying diagnosis.

## Discussion

The insula is a challenging neurosurgical structure from both diagnostic and surgical perspectives due to its complex anatomy, proximity to critical vasculature, and white matter tracts. Although glioma surgeons at high volume referral centers may see an abundance of what are ultimately pathologically proven to be insular gliomas, the present case illustrates the potential for other lesions in this region to mimic the classic expansile T2 hyperintensity of an insular glioma. Although arguments are sometimes made to observe small T2-hyperintense insular lesions to document growth prior to accepting the potential risks of surgery, evidence of contrast enhancement typically implies a more aggressive histology, discouraging conservative observation. We here illustrate a case wherein the opportunity for spontaneous resolution—perhaps facilitated by perioperative antibiotics at the time of biopsy—enabled a craniotomy to be avoided.

Advances in techniques and technologies including intraoperative MRI and awake mapping techniques have empowered safe resection of insular lesions with an acceptable risk-benefit profile for the vast majority of patients [5]. The importance of these developments is underscored by the survival benefit documented upon maximal safe resection of these lesions, which, untreated, can and eventually will undergo malignant transformation. Together with the substantial rate of nondiagnostic biopsies particularly in the case of insular and low-grade gliomas, oncologic neurosurgeons at high volume centers frequently advocate for prompt resection to optimize outcome [3]. For inoperable lesions, use of amino acid positron emission tomography-guided (PET-guided) biopsy has been shown to substantially increase the diagnostic yield, enabling targeting of the most metabolically active, and thus most likely high-grade region, even in non-enhancing tumors [6,7].

In retrospect, though our case with glioma-like imaging findings and seizure-like presentation was within typical limits for a neoplastic lesion, certain radiographic features did put abscess on the differential diagnosis. Specifically, a dark rim of T2 hypointensity surrounding the lesion, together with central restricted diffusion and radial stranding enhancement are findings consistent with abscess [8]. Conversely, the region of enhancement with central hypodensity within an expansile, anatomically bounded region of T2 hyperintensity and in a patient with new clinical symptoms, is a scenario all too reminiscent of progression from low to high grade glioma [9].

In terms of the generalizability of this case to the neurosurgical readership, our experience with this patient has not prompted us to change overall practice patterns in our institution; we continue to advocate for early surgical intervention for most patients with a significant clinical suspicion for glioma. Nevertheless, in the setting of increasingly favored surgery for insular gliomas at institutions worldwide, we felt this case to be an excellent and timely reminder of the imperative to critically scrutinize imaging findings and thoroughly explore all aspects of the clinical history. Moreover, in all such neuro-oncologic cases, a forthright discussion with the patient regarding diagnostic uncertainty must complement the surgical informed consent.

## Conclusions

The case we have presented herein illustrates the complexity of managing insular lesions and highlights the potential for alternate pathologies that can mimic insular glioma. In addition, it provides a humbling reminder that, even in the presence of seemingly pathognomonic imaging findings, a differential diagnosis of insular lesions must be thoughtfully considered in patient counseling and presurgical planning.
